# Unraveling Paracetamol Metabolism and Its Circadian Regulation: Insights from Tobacco Hairy Roots as a Model System

**DOI:** 10.3390/plants14172812

**Published:** 2025-09-08

**Authors:** Lucas G. Sosa Alderete, Mariana Vezza, Sabrina G. Ibañez, Peter Schroeder, Elizabeth Agostini, Melina A. Talano

**Affiliations:** 1 Departamento de Biología Molecular, Facultad de Ciencias Exactas, Físico-Químicas y Naturales, Instituto de Biotecnología Ambiental y Salud, INBIAS-CONICET, Universidad Nacional de Río Cuarto, Ruta Nacional 36 Km 601, Río Cuarto, Córdoba CP 5800, Argentina; mvezza@exa.unrc.edu.ar (M.V.); sibanez@exa.unrc.edu.ar (S.G.I.); eagostini@exa.unrc.edu.ar (E.A.); mtalano@exa.unrc.edu.ar (M.A.T.); 2 Unit Environmental Simulation, Helmholtz Zentrum München German Research Center for Environmental Health, 85764 Neuherberg, Germany; peter.schroeder@tum.de

**Keywords:** paracetamol removal, glutathione S-transferases, hairy roots cultures, circadian clock regulation

## Abstract

The increasing interest in the presence of contaminants of emerging concern (CEC) in aquatic environments has driven research into biological mechanisms capable of eliminating pharmaceutical compounds like paracetamol, considering different plant species as model systems. Thus, the use of hairy roots (HRs) has become an interesting tool. This study explores the ability of tobacco HRs to remove paracetamol, with an emphasis on elucidating the main metabolism steps and key enzymes involved in the green liver detoxification process, as well as the antioxidant response. The deepening of these aspects has been carried out through gene expression and biochemical analysis under circadian regulation. Our results reveal that HRs efficiently removed paracetamol (100 mg L^−1^) from the culture medium, achieving around 99% removal at ZT16 h (*Zeitgeber Time* 16). The early activation of antioxidant defense mechanisms, demonstrated by enhanced peroxidase (POD) activity and total antioxidant capacity (TAA) during the light phase, has been observed. Furthermore, glutathione S-transferase (GSTs) activity and glutathione (GSH) levels, potentially linked to paracetamol conjugation, were also assessed. Gene expression analyses confirmed GST gene upregulation in response to paracetamol treatment, with *GSTF6-like* and *GSTF8-like* maintaining circadian rhythms as in the control, and *GSTZ1-like* only displayed rhythmic expression upon treatment. Additionally, the modulation of core circadian clock genes (*NtLHY1* and *NtTOC1*) suggests that the plant response to paracetamol is tightly regulated by the circadian system. Together, these findings shed light on the complex molecular and biochemical mechanisms underlying paracetamol detoxification in tobacco HRs and underscore the significant role of circadian regulation in orchestrating these responses.

## 1. Introduction

The world is currently facing a significant challenge regarding the widespread decline in water quality. Over the last years, there has been increasing concern about so-called emerging contaminants (ECs), which are “those substances that are unregulated and could adversely affect the environment or whose effect is unknown” [1]. Pharmaceuticals have become particularly concerning due to their potential chronic toxicity and their contribution to the development of microbial resistance in humans and ecosystems. Given this issue, these compounds are being considered “contaminants of emerging concern” (CEC), a revised way to define them instead of ECs. This term has gained acceptance, especially in scientific and regulatory frameworks, to emphasize not only the novelty of these contaminants but also the growing recognition and the need for further attention based on the environmental impact and ecological threat [2].

In this context, paracetamol (also known as acetaminophen) has been widely used as an analgesic drug for relieving headaches, fever and body aches [3] and it is excreted as a component of human urine. In recent years, the concentration of paracetamol residue in domestic wastewater has increased due to the COVID-19 pandemic [4,5]. Furthermore, the manufacturing industry of paracetamol can also release this drug into the environment when it becomes expired or unneeded [6,7]. Due to its high stability, solubility, and hydrophilicity along with its increased consumption, paracetamol has been detected in surface waters, wastewater and drinking water worldwide [8]. Therefore, the removal of paracetamol and its metabolites or degradation products from wastewater, before their discharge into the environment, is essential to preserve environmental quality and to prevent harmful health effects on living organisms.

Paracetamol is known to be resistant to degradation by conventional biological and physico-chemical treatment processes in water or wastewater treatment plants [9]; thus, different strategies are being explored to manage the presence of this CEC. Among these, the use of plants for contaminant removal, commonly referred to as phytoremediation, has acquired considerable interest. Plants possess the ability to tolerate, transform, or degrade various pollutants, including CECs [4]. They achieve this through complex adaptive responses, including detoxification pathways and stress responses. The detoxification process, often referred to as the ‘green liver’ concept, consists of three main phases [10]. Phase I involves activation reactions, where oxidation/hydroxylation reactions are catalyzed by peroxidases (POD), hydroxylases, cytochromes P450 and other enzymes, followed by Phase II, where transferase enzymes such as glutathione S transferases (GSTs), UDP-glycosyl transferases (UDPGTs), among others, catalyze the conjugation of pollutants with hydrophilic molecules such as sugars, glutathione or amino acids. Finally, in Phase III, detoxification products are either compartmentalized (in the vacuole or cell wall) or secreted [11].

It is important to highlight that exposure to and uptake of xenobiotics may result in stress (or injury/damage) and the overproduction of reactive oxygen species (ROS) in plants, which cause damage to biomolecules and eventual cell death. To prevent ROS-related injury, plants developed antioxidant defense mechanisms consisting of enzymatic defense, including POD, glutathione reductases (GR) and others; as well as non-enzymatic scavengers (glutathione, ascorbate, tocopherols, among other). Therefore, the antioxidant response triggered by the presence of a contaminant, like paracetamol, is a suitable bioindicator of the ability of a plant to cope with stressful situations [12].

Among different plant systems commonly used for phytoremediation research, HRs cultures are considered a valuable tool. They show genotypic and phenotypic stability, high production of secondary metabolites, rapid and indefinite growth under in vitro conditions by subculturing on nutrient media in the absence of hormones, etc. [13,14]. Therefore, HRs cultures are considered interesting models for studying pollutants uptake, detoxification mechanisms and for identifying transformation products using diverse analytical methodologies [15,16]. In the case of paracetamol, Huber et al. [15] identified two major metabolic pathways in HRs of *Armoracia rusticana* (horseradish), leading to the synthesis of glutathione and a glucose conjugate [17].

The circadian clock (CC) is an endogenous timekeeping mechanism that allows organisms to anticipate and adapt to daily environmental changes [18]. In plants, the CC includes receptors that sense light or temperature and deliver this information to the core clock, which interprets environmental signals and operates within a negative feedback loop to maintain cycles of 24-h. Besides, the CC also has output responses including changes at the molecular, biochemical, and developmental levels [19]. Recently it has been suggested that environmental signals could be inhibited due to the presence of pollutants [20], causing differences in gene expression patterns to regulate diverse outputs. Thus, the CC performance under stressful situations would be essential to confer plant fitness and competitive advantage to survive and thrive under harsh conditions.

Given the prevalence and environmental hazard of paracetamol, this study aims to comprehensively (i) assess the ability of HRs cultures of *Nicotiana tabacum* (tobacco) to remove it and (ii) elucidate key aspects of the underlying detoxification mechanisms. Considering that this plant species is widely recognized as a model due to its fully sequenced genome and well-characterized gene functions and encoded protein, the results of the present study can provide valuable insights into the metabolic processes occurring in other plants. Additionally, paracetamol impact on stress defense mechanisms has been analyzed, with a particular focus on the role of CC genes in orchestrating an optimal response.

## 2. Results and Discussion

### 2.1. Analysis of Paracetamol Removal

Phytoremediation, recognized as a sustainable and environmentally friendly technology, has gained increasing attention as an interesting strategy for mitigating the impact of heavy metals, organic compounds and most recently CEC on the environment [16,21]. Assessing a plant ability to remove pollutants is a preliminary step in evaluating phytoremediation success (ref. [22] and references therein). This was the objective of the paracetamol treatments using *N. tabacum* HRs performed in this study.

**Figure 1:**
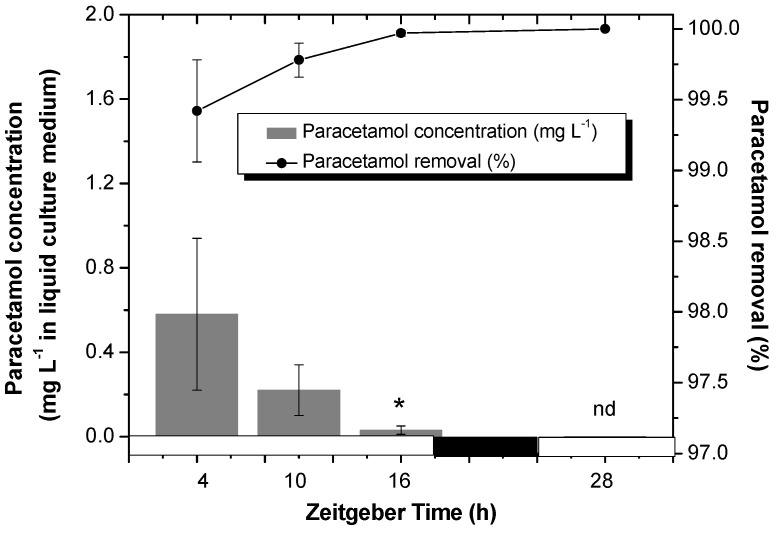
Residual paracetamol (mg L^−1^) detected in liquid culture medium (gray bars) and removal percentages (%) (black line) obtained for tobacco HRs treated with paracetamol. HRs were grown for two weeks under darkness conditions and from the third week they were synchronized with a photoperiod of 18 h light/6 h darkness. After 7 d of synchronization, paracetamol (100 mg L^−1^) was added to the MS medium at *Zeitgeber Time* (ZT) 4 and after 24 h of treatment, the samples were collected at different ZTs: 4, 10, 16 and 28. White and black bars represent light and dark phases, respectively. A clarification of “nd” reference as “not detectable” with our analytical method (HPLC). The data represent the average of three biological replicas (*n* = 3) ± standard error of the average. An asterisk (*) indicates significant difference at each time point (Duncan’s test, *p* ≤ 0.05).

In Figure 1, the remaining paracetamol concentration in culture medium is shown after different times of harvest (ZT4, ZT10, ZT16, and ZT28 h) (bars). The results were shown as percentage of paracetamol removal (lines) considering 100 mg L^−1^ as initial concentration.

The results suggest that the tobacco HRs system is highly efficient in removing paracetamol from the liquid culture medium, with significant reduction already observed after the first hours of incubation/treatment. It is noteworthy that in the first 4 h of treatment only a small concentration of paracetamol remains in the culture medium (around 0.6 mg L^−1^), reaching a removal percentage value of 99.4%. At ZT28, paracetamol was no longer detected (nd), indicating complete removal (100%).

The high removal efficiency found for paracetamol, even for concentrations as high as 100 mg L^−1^, is a fact of great importance considering those findings reached by Omotola et al. [23]. They found phytotoxic effects in *Allium cepa*, such as reduction in the germination percentage and in the rate of root growth, as well as apoptotic markers (DNA fragmentation), even at paracetamol concentrations as low as 0.1 mg L^−1^. Also, Badar et al. [17] using spinach (*Spinacia oleracea* L.) plants and paracetamol concentrations from 50 to 200 mg L^−1^ found significantly increased oxidative stress and DNA damage. Moreover, this paracetamol exposure led to the activation of stress-responsive genes, revealing its substantial molecular toxicity and highlighting the necessity for reliable methods to eliminate it from the environment. Although the paracetamol concentrations used in this study may exceed typical environmental pollution levels, the results provide valuable insights into the plant defense mechanisms against pharmaceutical contaminants. This knowledge is particularly relevant for understanding plant responses in scenarios of accidental or deliberate contamination, thereby contributing to broader discussions on phytotoxicity and environmental safety.

Consistent with our results, Kotyza et al. [24] demonstrated that *Armoracia rusticana* L. HR was capable of completely removing paracetamol at concentrations of 90 and 180 mg L^−1^ over an 8-day period, achieving approximately 50% removal within the first 24 h. Additionally, they effectively managed four consecutive treatment cycles, each with 150 mg L^−1^ of paracetamol. Based on these results, the authors proposed that recycling assays employing *A. rusticana* hairy roots could serve as a model for continuous-flow systems (rhizofiltration) for paracetamol remediation.

### 2.2. Biochemical Response to Paracetamol Treatment

#### 2.2.1. POD Activity

**Figure 2:**
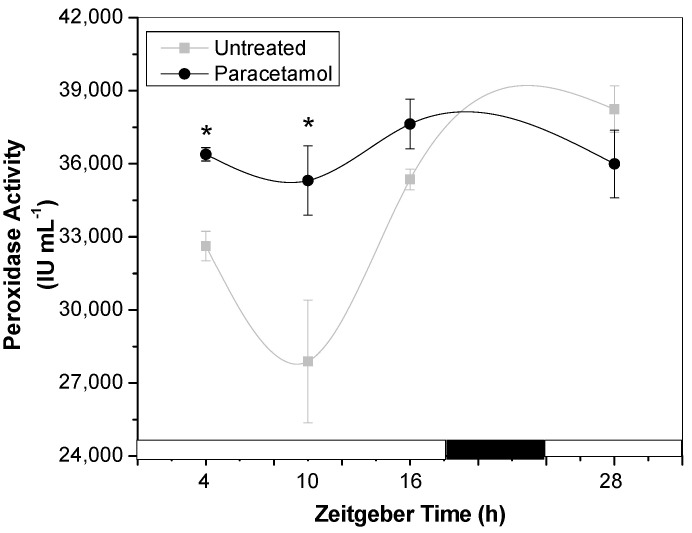
Activity of total peroxidase (POD) determined in tobacco HRs under control conditions (gray line) and after treatment with paracetamol (black line). HRs were grown for two weeks under darkness conditions and from the third week they were synchronized with a photoperiod of 18 h light/6 h darkness. After 7 d of synchronization, paracetamol (100 mg L^−1^) was added to MS medium at *Zeitgeber Time* (ZT) 4 and after 24 h of treatment, samples were collected at different ZTs: 4, 10, 16 and 28. White and black bars represent light and dark phases, respectively. The data represent the average of three biological replicas (*n* = 3) ± standard error of the average. The results are expressed as International Units per milliliter (IU mL^−1^). * indicates significant difference between control and treated HRs in each time point (Duncan test, *p* ≤ 0.05).

Class III plant peroxidases (PODs) belong to a large multigene family that participates in various physiological processes, including defense responses, ROS metabolism, hormone biosynthesis and degradation, as well as cell wall formation and maintenance. The enzymes share similar three-dimensional structures and a common catalytic mechanism for hydrogen peroxide degradation. Numerous studies have investigated plant antioxidant mechanisms, including POD, as a useful strategy to develop transgenic crop plants with increased tolerance to multiple stresses [25]. Besides, POD are also key enzymes involved in phase I during the xenobiotic metabolism [26]. Therefore, this study examined the effect of paracetamol treatment on POD activity in synchronized tobacco HRs. The possible regulation by CC on POD performance during this stressful condition was also discussed. As it is shown in Figure 2, in untreated HRs, POD activity showed an oscillatory performance with high activity both at the beginning and at the end of light phase (ZT4, ZT28 and ZT16), with ZT10 being the time of lowest POD activity. Likewise, untreated-HRs conserved a period shorter than 24 h (ultradian period) as had been previously observed [27].

Tobacco HRs exposed to paracetamol exhibited significantly elevated POD activity at ZT4 and ZT10 compared to control HRs. This observation is consistent with findings by Bartha et al. [28], who reported notable increases in POD activity, particularly in leaf tissues, after 24 h of paracetamol exposure. Additionally, previous studies from our research group have demonstrated similar increases in POD activity in tobacco HRs treated with other contaminants of emerging concern (CECs), such as antibiotics and 17α-ethinyl estradiol [16].

#### 2.2.2. GST Activity

To assess the effects of paracetamol treatment on the activity of GSTs, key enzymes in phase II of the metabolism of xenobiotic compounds, two substrates were employed: 1-chloro-2,4-dinitrobenzene (CDNB) and p-nitrophenyl acetate (pNpA).

**Figure 3:**
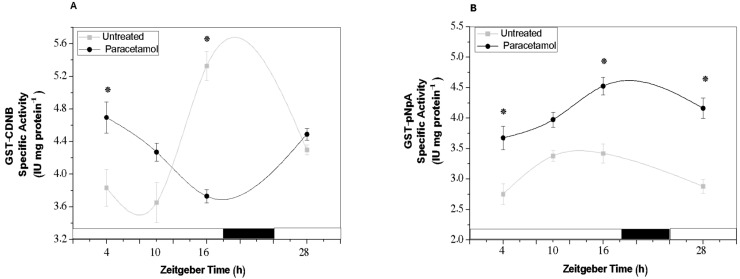
Glutathione S-transferase (GST) activity evaluated in tobacco HRs under control conditions (gray line) and after treatment with paracetamol (black line). HRs were grown for two weeks under darkness conditions and from the third week they were synchronized with a photoperiod of 18 h light/6 h darkness. After 7 d of synchronization, paracetamol (100 mg L^−1^ was added to MS medium at *Zeitgeber Time* (ZT) 4 and after 24 h of treatment, samples were collected at different ZTs: 4, 10, 16 and 28. The enzymatic activity was assayed with two substrates: CDNB (**A**) and pNpA (**B**). White and black bars represent light and dark phases, respectively. The data represent the average of three biological replicas (*n* = 3) ± standard error of the average. The results are expressed as specific activity (IU mg protein^−1^). * indicates significant difference between control and treated HRs in each time point (Duncan test, *p* ≤ 0.05).

Under control conditions, the GST activity profiles for the conjugation of both substrates displayed a circadian rhythm, with a single peak of increased activity at ZT16 (end of the light phase). The activity peak was predominantly observed with CDNB as the substrate, while for pNpA, it occurred between ZT10 and ZT16 (Figure 3A,B). Notably, enzyme activity profiles varied depending on the substrate used and were differentially influenced by paracetamol treatment. In the case of CDNB, HR cultures exhibited an anti-phase activity pattern compared to the control, with the highest GST activity detected at ZT4 and ZT10, followed by a marked decrease at ZT16. By contrast, when pNpA was used as the substrate, GST activity in paracetamol-treated HRs was significantly elevated at nearly all time points analyzed. This activity maintained a similar enzyme profile as the control, with maximum activity observed towards the end of the light phase (ZT16). Bartha et al. [28] have demonstrated differences in GST activity for various substrates. Generally, CDNB is considered a standard substrate for GST determination, being conjugated at higher rates than pNpA, as it was observed in our results (Figure 3A,B). Based on these results, GST activity could be regulated by the CC in synchronized tobacco HRs. However, the presence of paracetamol affects such regulation, mainly when CDNB was used as a substrate. Therefore, according to these results, we can suggest that paracetamol not only modifies GST activity, but also induces a synchronization signal by resetting the circadian performance of the enzyme. Several reports have shown different behaviors (significant increase and decrease) in GST activity under xenobiotic exposure, such as paracetamol, diclofenac, among others, highlighting the role of the GSTs and GSH during the xenobiotic detoxification [28,29,30,31,32]. However, little is known about the effect of paracetamol on circadian modulation of GST and even less is known about the direct effect on the clock gene function. In line with our findings, mainly in untreated HRs, Gallé et al. [33] also found circadian rhythms in GSTs activity as well as in the expression of selected GST genes of tomato plants. Our results showed that under paracetamol exposure, tobacco HRs conserved a circadian performance of GST activity maintaining the same phase or an anti-phase depending on the substrates employed, pNpA and CDNB, respectively.

#### 2.2.3. TAA and GSH Content

Plants have evolved key physiological and metabolic mechanisms destined to alleviate environmental stresses such as drought, cold, salinity, inorganic and organic pollutant toxicity. During stress, harmful by-products, such as ROS, are produced. To counteract excessive ROS production and oxidative stress, plants rely on a tightly regulated antioxidant system composed of both enzymatic and non-enzymatic components. These elements work in concert to balance ROS generation and scavenging, thereby maintaining ROS homeostasis and protecting essential macromolecules such as lipids, proteins, and DNA from oxidative damage [34]. Thus, in the present work, TAA was assayed as a measure of non-enzymatic antioxidant components.

**Figure 4:**
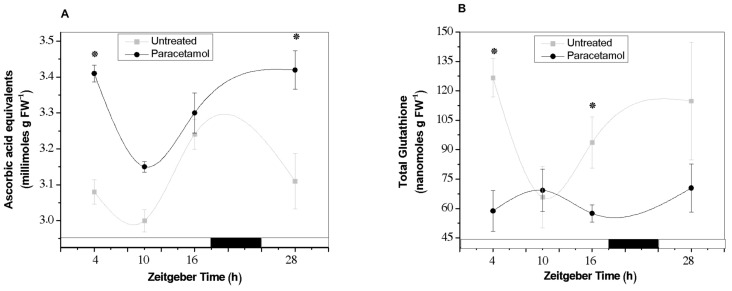
Total antioxidant activity (TAA) (**A**) and total GSH content (**B**) were determined in tobacco HRs under control (gray line) conditions and after treatment with paracetamol (black line). White and black bars represent light and dark phases, respectively. The data represent the average of three biological replicas (*n* = 3) ± standard error of the average. * indicates significant difference between control and treated HRs in each time point (Duncan test, *p* ≤ 0.05). TAA is expressed as millimoles ascorbic acid equivalents (AAE) per g fresh weight (millimoles AAE g FW^−1^) (**A**). Total GSH is expressed as nanomoles per G fresh weight (nmoles g FW^−1^) (**B**).

Our results showed that TAA seems to conserve a circadian profile across the analyzed times under control conditions, with low antioxidant capacity from ZT4 to ZT10 but showing the highest value of TAA at ZT16 (end of the light phase). However, when tobacco HRs were exposed to paracetamol, the circadian oscillation of TAA was modified, showing significant increases mainly at the beginning of the light phase, ZT4 and ZT28 (Figure 4A). According to these results, paracetamol-treated HRs quickly increase their non-enzymatic antioxidant capacity, mitigating stress induced by this CEC. In this context, it was also interesting to evaluate total GSH content since it could provide important information on the effect of paracetamol on the GSH pool. As it is known, GSH not only works as a non-enzymatic antioxidant, but GSH (reduced form) is also the primary substrate of the detoxifying GSTs previously analyzed. Under control conditions, the highest total GSH levels were detected at the beginning of the light phase (ZT4 and ZT28) as well as at the end of the light phase (ZT16) (Figure 4B). By contrast, in paracetamol-treated HRs no significant changes were detected in the total GSH content across all analyzed times. However, in comparison to untreated HRs, the total GSH was significantly decreased mainly at ZT4 and ZT16. According to these findings, the decrease in GSH levels could be directly related to the significant increases of GST activity detected in paracetamol-treated HRs. These increases were mainly observed at ZT4 and ZT16, using CDNB (at ZT4, Figure 3A) or pNpa as substrate (at ZT4 and ZT16, Figure 3B), indicating that GSH was probably used during paracetamol GS-conjugation catalyzed by GSTs. In line with our results, Sousa et al. [32,35] found low GSH content and significant increases in the GST activity in roots of *Solanum lycopersicum* L. plants treated with 5 mg L^−1^ diclofenac (DFC). These authors emphasized that GSH-mediated DCF detoxification carried out by GSTs was the main mechanism activated in tomato root. Moreover, previous reports have shown that not only organic pollutants can affect the GSH content but also heavy metals exposure induced significant decrease in the GSH and GSSG levels in *Picea abies* L. suspension culture and *Glycine max* L. roots [29,36]. Furthermore, consistent with our results, it was observed that GSH levels were higher during the light phase than during the night phase in several plant species, suggesting that GSH contents may be affected by diurnal regulation (reviewed by Gallé et al. [37]).

#### 2.2.4. TBARs Content

**Figure 5:**
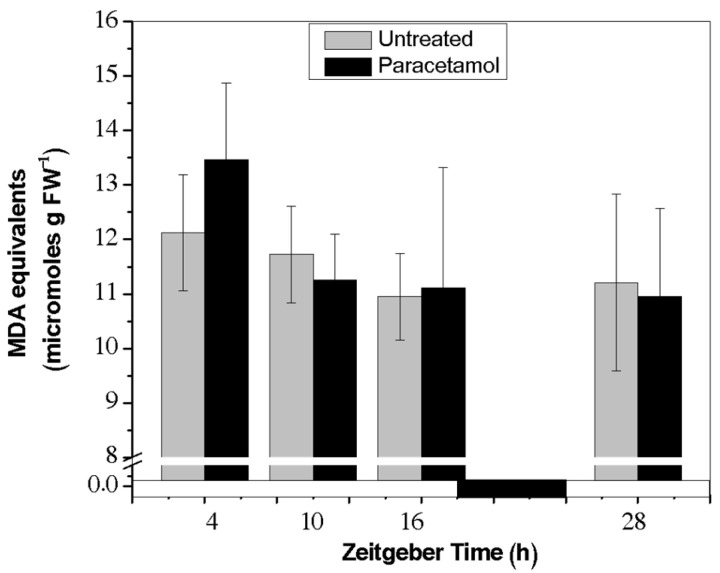
Reactive thiobarbituric acid substances (TBARs) content (μmoles MDA equivalents g^−1^) in tobacco HRs under control conditions (gray bar) and after treatment with paracetamol (black bar) at different times (ZT4, ZT10, ZT16 and ZT28). The data represent the average of three biological replicas (*n* = 3) ± standard error of the average. White and black bars represent light and dark phases, respectively.

Excess ROS accumulation accelerates membrane lipid peroxidation and consequently MDA content [38]. The results obtained in this study demonstrated that ROS production and scavenging systems are in a dynamic balance, as no significant differences were observed comparing untreated and treated HRs at any of the investigated time points (Figure 5). As mentioned above, in presence of paracetamol tobacco HRs were able to significantly induce the antioxidant defense reported mainly by POD activity (Figure 2) and TAA (Figure 4A) which attenuated the degree of membrane lipid peroxidation, frequently caused by ROS accumulation. Several studies have focused on oxidative damage on plant membrane lipids, after different CEC exposure. For instance, *Medicago sativa* plants showed a significant oxidative effect on the root lipids after treatment with a mixture of pharmaceutical compounds (diclofenac, sulfamethoxazole, trimethoprim, 17a-ethinylestradiol) [39]. Bisphenol A treatment also induced H_2_O_2_ and O_2_^−^ accumulation, leading to increases in membrane lipid peroxidation in soybean seedling roots [40]. Other reports, in different biological models (shell and mussel), have shown lipid peroxidation after several days of ibuprofen treatment [41,42]. Moreover, in mammals, paracetamol treatment increased lipid peroxidation and over-production of ROS and decreased GSH levels in the livers of mice [43]. Based on our results, paracetamol treatment did not cause oxidative damage on the membrane lipids, due to an efficient functioning of the antioxidant defense system in charge of mitigating ROS overproduction.

### 2.3. Impact of Paracetamol Treatment on Gene Expression

#### 2.3.1. GST Gene Expression

Taking into account the role of GST enzymes in the detoxification of organic compounds, we evaluated the effect of paracetamol treatment on the expression of selected GST genes. Additionally, we analyzed the implication of the CC on the regulation of these expression patterns.

For the selection of GST genes, we considered genes that have been shown to respond to other xenobiotic compounds [44,45], as well as those whose expression was under the control of the CC (Tables S1 and S2).

**Figure 6:**
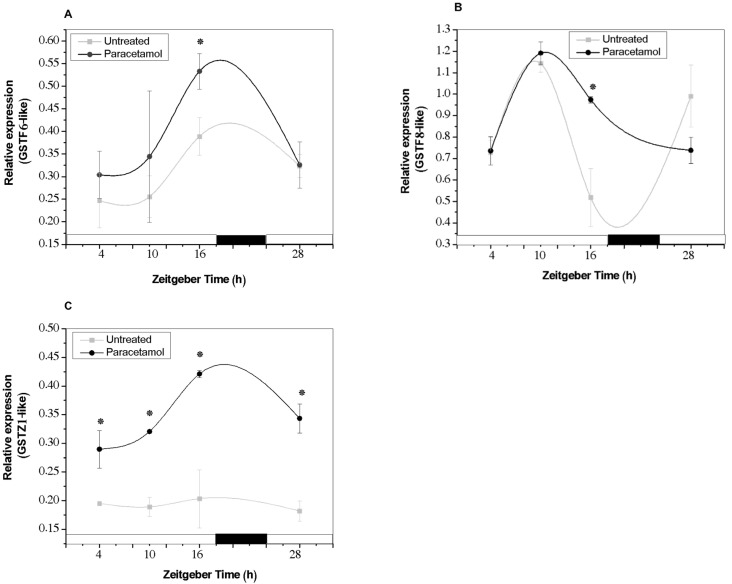
Relative expression of the *GSTF6-like* (**A**), *GSTF8-like* (**B**) and *GSTZ1-like* (**C**) genes in tobacco HRs under control conditions (gray line) and after treatment with paracetamol (black line) was evaluated. White and black bars represent light and dark phases, respectively. HRs were grown for two weeks under darkness conditions and from the third were synchronized with a photoperiod of 18 h light/6 h darkness. After 7 d of synchronization, paracetamol (100 mg L^−1^) was added to the MS medium at *Zeitgeber Time* (ZT) 4 and after 24 h of treatment, the samples were collected at different ZTs: 4, 10, 16 and 28. The data represent the average of three biological replicas (*n* = 3) ± standard error of the average. * indicates significant difference between control and treated HRs in each time point (Duncan test, *p* ≤ 0.05).

As shown in Figure 6A,B, the *GSTF6-like* and *GSTF8-like* genes exhibited circadian expression profiles under control conditions, with maximum expression peaks occurring during the light phase at ZT16 and ZT10, respectively. In contrast, the expression profile of the *GSTZ1-like* gene showed no significant changes throughout the 24-h cycle (Figure 6C). When tobacco HRs were treated with 100 mg L^−1^ paracetamol, a significant induction in the expression of these genes was observed, mainly in *GSTZ1-like*. This gene was induced at all analyzed times, with a maximum peak at ZT16 (Figure 6C). Paracetamol treatment did not modify the phases of the circadian expression profiles of *GSTF6-like* and *GSTF8-like*; however, the expression of both genes increased towards the end of the light phase (ZT16). Previous research conducted by our group showed that the *GSTF8-like* gene also shaped a circadian expression profile under control conditions; however, under phenol treatment, the circadian expression was completely lost, with a significant increase at all analyzed time points [46]. Besides, it was observed that, although these genes maintained circadian expression patterns in *N. tabacum,* their peak expression times were different compared to those observed in their orthologous genes in *A. thaliana* (Table S1). Thus, circadian expression profiles of these genes could differ depending on plant species and tissue, as observed in other reports ([36] and references cited therein). In this context, a search for circadian cis-regulatory elements in promoter region of selected GST genes revealed the presence of at least one circadian cis-regulatory sequences (CCRS) of 10–11 nucleotides with the following consensus sequence CAANNNNATC (where N corresponds to any nucleotides) (Table S2). These CCRS would be essential for the circadian modulation of these selected GST gene expression. In agreement with our findings, circadian rhythms in both GST activity and mRNA accumulation of selected GST genes were also found in *Solanum lycopersicom* L. [33]. Moreover, the key role of light condition (quality, duration and intensity of illumination) which is able to induce a diurnal control both in GST activity and in the performance of GST gene expression reviewed by [37] has been observed in various plant species.

#### 2.3.2. Clock Gene Expression

It is known that the CC functions as the master regulator of plant gene expression, controlling over 30% of the transcriptome [47]. These genes are involved in many relevant physiological processes, such as flowering time, photosynthesis, gas exchange, redox state and stress responses, among others [46,48,49]. Indeed, circadian clock disruption or mutations in its components lead to changes in the sensitivity to several adverse conditions [50].

**Figure 7:**
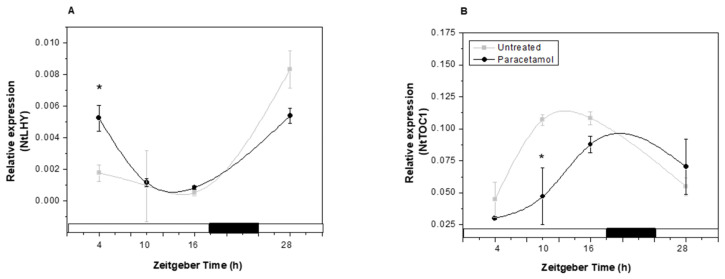
Relative expression of clock gene: *NtLHY* (**A**) and *NtTOC1* (**B**) in tobacco HRs under control conditions (gray line) and after treatment with paracetamol (black line) was analyzed. HRs were grown for two weeks under darkness conditions and from the third were synchronized with a photoperiod of 18 h light/6 h darkness. After 7 d of synchronization, paracetamol (100 mg L^−1^) was added to the MS medium at *Zeitgeber Time* (ZT) 4 and after 24 h of treatment, the samples were collected at different ZTs: 4, 10, 16 and 28. White and black bars represent light and dark phases, respectively. The data represent the average of three biological replicas (*n* = 3) ± standard error of the average. * indicates significant difference between control and treated HRs in each time point (Duncan test, *p* ≤ 0.05).

In the present study, paracetamol treatment modified the expression profiles of two key CC genes, *NtLHY* and *NtTOC1*. While paracetamol exposure did not alter the phase of the expression profiles of these genes, it induced both an increase and a decrease in *NtLHY* expression at ZT4 and ZT28, respectively (Figure 7A). Regarding *NtTOC1* expression profile, a 6-h delay in its accumulation peak was detected in paracetamol-treated-HRs compared to the control (Figure 7B), being the expression peaks observed at ZT10 and ZT16 in untreated and paracetamol-treated HRs, respectively. In this context, few reports have explored the effect of organic pollutants on the circadian clock performance. For instance, phenol treatment also produced down-regulation in the expression of CC genes and xenobiotic metabolism-related genes in tobacco HRs [20,46]. Based on the available literature, little is known about the impact of CEC exposure on plant circadian clocks, and even less about the physiological events under circadian control. Thus, our results may represent one of the first findings that explore the physiological response to paracetamol treatment, evaluating the capacity to remove it from the culture medium and the involvement of circadian clocks during the detoxification process and stress response.

## 3. Material and Methods

### 3.1. Reagents

Paracetamol (Acetaminophen) was purchased from Sigma-Aldrich (St Quentin Fallavier, France), with a purity of >95%. High-purity (>98.0%) solvent (methanol) and deionized water were used for HPLC. Paracetamol stock solution used both for treatment and for standard calibration curve was prepared with deionized water at a concentration of 12,080 mg L^−1^.

### 3.2. Biological Material and Growth Conditions

HR cultures of *N. tabacum* (var Wisconsin) were successfully obtained by infecting leaf explants, from sterile tobacco seedlings, with *Agrobacterium rhizogenes* LBA 9402, as reported by Sosa Alderete et al. [51]. They were regularly subcultured (each 25–30 days) and maintained in Murashige and Skoog (MS) liquid medium [52] in an orbital shaker (70 rpm) at 25 ± 2 °C in dark [51]. HRs cultures of 21 days were synchronized during 7 days under a light/dark photoperiod (18 h light/6 h dark, 60–100 μmoL m^−2^ s^−1^ of cool white fluorescent light) [46]. It is important to mention that the term *Zeitgeber Time* (ZT) (literally, “*time giver*” or “*time cue*”) is a standardized way of expressing time in circadian biology. It refers to the time elapsed since the onset of light, which is considered the strongest zeitgeber for all circadian systems and the cue that synchronizes the biological clock [53].

### 3.3. Paracetamol Treatments

After the synchronization period, HRs were treated with a sterilized paracetamol solution (100 mg L^−1^) at *Zeitgeber Time* (ZT) ZT4 (light phase), considering ZT0 as the beginning of the light phase. Liquid medium and HRs were harvested after 24 h of treatment at ZT4, ZT10, ZT16, and ZT28, all during the light phase. Liquid medium was stored at −20 °C and then used to analyze residual paracetamol; while HRs were washed with deionized water, dried with filter paper, macerated in liquid N_2_ and stored at −80 °C for further studies. For control HRs an equal volume of sterilized water, in place of the paracetamol solution was added.

### 3.4. Paracetamol Removal Analysis

Liquid medium aliquots were filtered with a nylon membrane filter (0.45 µm pore size, 25 mm diameter) to remove all solid particles. The paracetamol concentration was estimated by HPLC analysis. An LC system (LC-NetII/ADC interface) equipped with a Jasco PU-4080i pump and a Jasco MD-4010 photodiode array detector (Jasco, Easton, MD, USA) was used. Manual sample injection (25 µL) was carried out. The detector was set at 245 nm. Methanol/KH_2_PO_4_ 10 mM (pH 4.7) mobile phase in the ratio 80/20 *v*/*v* was used in isocratic elution at a flow rate of 1 mL min^−1^. For separation a Chrom-Clone Phenomenex (Torrance, CA, USA) C18 column (250 mm length; 6 mm inner diameter; 5 µm; 100 Å) was used, joined to a guard column (KJ0-4282, Phenomenex, Torrance, CA, USA; C18) to safeguard the analytical column. For paracetamol, mean retention time of 8.45 ± 0.10 min was recorded. The quantitative analysis was performed with external standardization. Peak areas were integrated automatically using the ChromNAV 2.0 software program. The results were expressed as residual paracetamol (mg L^−1^) detected in liquid culture medium and as removal percentages (%), where a paracetamol solution (100 mg L^−1^) incubated in the same medium and under identical experimental conditions was considered 100%.

### 3.5. Biochemical Analysis

#### 3.5.1. Determination of POD Activity

HRs (200 mg) were homogenized with 0.6 mL of 50 mM AcNa/AcH (sodium acetate/acetic acid) pH 5 buffer solution containing 1 M KCl. Afterward, the samples were centrifuged at 10,000 rpm for 10 min. The obtained supernatant was considered a soluble extract of total POD. A mixture reaction containing 100 mM AcNa/AcH buffer pH 5.5, 0.63 mM *o*-dianisidine, 0.5 mM H_2_O_2_ and 10 μL of diluted 1/20 crude POD extract was prepared. In the presence of POD and H_2_O_2_, *o*-dianisidine undergoes oxidation to form a colored product, [bis(3,3′-dimethoxy-4-amino)azo-biphenyl], which is continuously monitored at 470 nm using spectrophotometry (ε = 11.3 mM^−1^) [51]. One unit of enzyme (U) was defined as the amount of enzyme able to produce 1 μmol of product in 1 min in the conditions described.

#### 3.5.2. Determination of GST Activity

To obtain the soluble enzyme fraction, a methodology described by Schröder et al. [54] was carried out. Frozen plant material was powdered and extracted with buffer (0.1 M Tris/HCl pH 7.8, 5 mM EDTA, 5 mM dithioerythritol DTE, 1% Nonidet P40, 1% insoluble polyvinylpyrrolidone PVP K90), homogenized and centrifuged for 30 min at 20,000 rpm. Proteins in the obtained crude extract were precipitated by addition of ammonium sulphate in two steps of 40% and 80% of saturation, respectively. The homogenate was centrifuged after each step and the pellet was finally resuspended in 25 mM Tris/HCl buffer pH 7.8. This step was followed by desalting the extracts with Sephadex PD-10 columns (Pharmacia, Freiburg, Germany). GST activity was measured using spectrophotometric assays following the protocol described by Schröder et al. [54]. Two substrates were employed for this purpose: 1-chloro-2,4-dinitrobenzene (CDNB, ε340 nm = 9.6 mM^−1^) and p-nitrophenyl acetate (pNpA, ε310 nm = 1.9 mM^−1^). Protein concentration was determined according to [55] using serum albumin as a standard.

#### 3.5.3. Total Antioxidant Activity (TAA)

TAA was assessed based on the free radical scavenging activity of DPPH (1,1-diphenyl-2-picrylhydrazyl) as it was described by Brand-Williams [56] with minor modifications [57]. For this, frozen HRs samples (75 mg) were homogenized in 1 mL of 50% (*v*/*v*) methanol, kept on ice for 90 min and then centrifuged at 10,000 rpm and 4 °C for 15 min. The resulting supernatant (50 μL) was mixed with 60 μM DPPH methanol solution and incubated in darkness for 30 min at room temperature. Absorbance was then measured at 515 nm. A calibration curve was prepared using ascorbic acid, and the antioxidant capacity of the HR extracts was informed as millimoles of ascorbic acid equivalents (AAE) per gram of fresh weight (mmol AAE g FW^−1^).

#### 3.5.4. Evaluation of Glutathione (GSH) Concentration

To determine total GSH content, frozen HRs samples (0.15 g) were homogenized in 5% sulfosalicylic acid (1 mL) and centrifuged at 10,000 rpm, 4 °C for 15 min. GSH was determined through the method described by Anderson [58]. For this, a reaction mixture was prepared using 143 mM sodium phosphate buffer (pH 7.5), 6.3 mM EDTA, 0.248 mg mL^−1^ NADPH and 6 mM DTNB [5,5′-Dithiobis (2-nitrobenzoic acid)], and incubated at 30 °C for 10 min. The reaction was started by adding the previously obtained supernatant (30 µL) and the GSH reductase enzyme (GR) (1.3 U). GSH content was plotted against changes in absorbance at 412 nm. Reference standards of 5–60 μM GSH were prepared and analyzed in the same way to obtain a calibration curve.

#### 3.5.5. Evaluation of Thiobarbituric Acid Reactive Substances (TBARs) Content

The TBARs content, an indicator of lipid peroxidation, was measured and expressed as malondialdehyde (MDA) equivalents. The assay followed the methodology described by Heath and Packer [59]. Lipid peroxides were quantified based on their reaction with thiobarbituric acid (TBA), forming a colored complex whose absorbance was measured. The results were expressed as μmoles of MDA equivalents/gram of fresh weight using an extinction coefficient of 155 mM^−1^ cm^−1^.

### 3.6. Analysis of GST and Clock Genes

#### 3.6.1. Identification of Putative GST Genes in *N. tabacum*

The putative GST genes involved in phase II of the metabolism of xenobiotic compounds were selected according to previous studies that confirmed their up-regulation under stress conditions, mainly in the model plant *Arabidospis thaliana* [44,45,60,61]. Another criterion for the selection of GST genes from *A. thaliana* was the presence of a circadian expression pattern. This analysis was carried out using the Diurnal page (http://diurnal.mocklerlab.org/diurnal_data_finders/new, accessed on 6 December 2017), which made it possible to determine the peak expression of each of the selected GST genes at a given ZT. After identifying these GST genes in *A. thaliana*, we proceeded to identify homolog genes in the *N. tabacum* genome. For this, the corresponding amino acid sequences of *A. thaliana* obtained from TAIR (https://www.arabidopsis.org) database were used as queries in BLAST (BLAST+ version 2.7.1) searches (TBLASTN tool) against the *N. tabacum* genome TN90, by using the National Center of Biotechnology Information (NCBI) database (https://blast.ncbi.nlm.nih.gov/Blast.cgi, accessed on 3 July 2024). Tobacco nucleotide sequences with e-values close to zero were selected for further analysis (Supporting Information Table S1).

#### 3.6.2. Identification of Circadian Clock Cis-Elements in the Promoter Regions of GST Genes

The promoter regions of putative GST genes in *N. tabacum* were screened for circadian-related cis-regulatory elements using the PlantCARE database [62]. For this purpose, promoter regions of putative GST genes, including −1.5 kb sequences (upstream) and +20 bp (downstream) from the transcription start site were analyzed (Supporting Information Table S2).

#### 3.6.3. qPCR Analysis of GST and Clock Genes Expression

From the in silico analysis, three GST putative genes were selected which were designed as *GSTF6-like*, *GSTF8-like* and *GSTZ1-like* based on the similarity with the corresponding genes of *A. thaliana*. The expression profiles of these genes and key clock genes, *LATE ELONGATED HYPOCOTYL* (*NtLHY*) and *TIMING OF CAB EXPRESSION 1* (*NtTOC1*), both belonging to the central circadian clock-loop, were analyzed by qPCR after paracetamol treatment. For that, total RNA was extracted using Trizol reagent (Invitrogen) according to the supplier instructions. Absorbance readings at 260 and 260/280 nm were measured in order to evaluate concentration and purity of total RNA. Then, total RNA samples (1 μg) were treated with DNase (Promega, Madison, WI, USA) and then converted to cDNA by reverse transcription (MMLV Promega) using Random primer (100 ng μL^−1^) (Biodynamics, Akron, OH, USA). For amplification by qPCR, a mix was prepared with 1 μL of the cDNA, 400 nM forward-reverse primers and 7.5 μL of Itaq Universal SYBR Green supermix (Bio Rad, Hercules, CA, USA) in a total volume of 15 μL-PCR. This reaction was carried out considering the following cycling conditions: initial denaturation at 95 °C for 3 min, followed by 40 cycles of 95 °C for 15 s, 60 °C for 30 s, and 72 °C for 30 s. Standard curve linearity and amplification efficiency were optimized for each primer pair. The normalized expression (NE) was calculated using the following formula: NE = 2^−ΔCt^, where ΔCt = Ct experimental − Ct normalizer [63]. Elongation factor 1 (*NtEF1*) gene was used as a reference gene, previously identified as stably expressed under many developmental stages and stress situations [64]. It is important to note that *N. tabacum* species are allotetraploid, therefore, all primers employed in this work were designed to be specific to a single gene. For this purpose, integrated DNA technologies tools (https://www.idtdna.com) were used (Primers list is shown in Supporting Information Table S3).

### 3.7. Data Analysis 

Results were obtained from at least three independent biological replicates, each evaluated in two technical replicates. Then the data from all the determinations were statistically processed by analysis of variance (ANOVA), using the STATISTICA 6.0 software and applying the Duncan’ Test (*p* < 0.05).

## 4. Conclusions

Based on our findings, we can assert that nearly the entire initial concentration of paracetamol (100 mg L^−1^) was removed from the culture medium by tobacco HRs. Through this research, some aspects related to paracetamol metabolism in plant tissues and responses based on gene expression patterns and the biochemical data of key elements were elucidated. In this model plant, there appears to be the early activation of both enzymatic and non-enzymatic antioxidant systems, as indicated by increased POD activity and total antioxidant capacity (TAA) during the early light phase. POD activity may also play a pivotal role in Phase I of paracetamol detoxification by catalyzing oxidation reactions, which are subsequently followed by Phase II conjugation processes mediated by glutathione S-transferases (GSTs). This process would be likely complemented by GST-mediated detoxification, as suggested by elevated GST activity and concurrent reductions in GST protein levels at corresponding times, reinforcing the hypothesis that GSH is being used as substrate by GSTs to conjugate paracetamol.

Moreover, the upregulation of GST gene expression was induced by paracetamol exposure, maintaining the same circadian expression patterns for *GSTF6-like* and *GSTF8-like* genes, and even inducing rhythmic expression in *GSTZ1-like*. These findings suggest that paracetamol not only engages detoxification pathways but also may influence circadian regulation. The observed modulation of core circadian clock gene expression further supports the idea that the plant response to this CEC may be under circadian control.

Finally, this work provides new insights for using HRs as a feasible strategy for the efficient treatment of water polluted with paracetamol. Furthermore, the use of bioreactors specially designed for HRs growth could enable the phytoremediation process to be carried out on a large scale.

## Data Availability

The datasets used and/or analyzed during the current study are available from the corresponding author on reasonable request.

## Supplementary Materials

The following supporting information can be downloaded at: https://www.mdpi.com/article/10.3390/plants14172812/s1, Table S1: Identification by BLASTX analysis of GSTs genes; Table S2: Identification of regulatory elements under circadian control; Table S3: Primers list used for quantitative real-time PCR expression studies.
