# Supplementary material for: Unraveling Paracetamol Metabolism and Its Circadian Regulation: Insights from Tobacco Hairy Roots as a Model System
Source: Plants (Basel). 2025 Sep 8;14(17):2812. doi: 10.3390/plants14172812 (PMC12430747; doi:10.3390/plants14172812)
Supplement: Supplementary file 1 [file plants-14-02812-s001.zip › plants-3831974-supplementary.pdf]

Supplementary material

Table S1: Identification by BLASTX analysis of GSTs genes potentially involved in metabolism of xenobiotic compounds (MXC) in *Nicotiana tabacum* from *Arabidospsis thaliana* protein sequences. The E-value and identity obtained by BLASTX corresponds to the local alignment between the nucleotide sequences found in tobacco and the protein sequences of *A. thaliana.* One of the selection criteria for the *A. thaliana* proteins used in BLASTX was the presence of a circadian expression pattern in their respective genes. This analysis was carried out using the Diurnal page (http://diurnal.mocklerlab.org/diurnal_data_finders/new), which made it possible to determine the peak expression of each of the selected *A. thaliana* genes at a given *Zeitgeber* time (ZT).

Genes family	*Name of gene in N. tabacum*	*Gene identity* (ID) *in N. tabacum*	*Name of gene in A. thaliana*	*Gene identity* (ID) in *A. thaliana*	Blast-E value	Identity (%)	Analysis of oscillation by Diurnal/peak expression (h)	Plant specific class
GSTs	▪ *GSTF6-like*	107794811	*GSTF6*	AT1G02930	2e-73	55	zt20	Phi
	▪ *GSTF8-like*	107795177	*GSTF8*	At2G47730	1E-89	60	zt4	Phi
	▪ *GSTZ1-like*	107828152	*ATGSTZ1*	AT2G02390	9E-103	66	zt20	zeta

Table S2: Identification of regulatory elements under circadian control in the promoter region of selected GSTs genes in tobacco. PlantCare software was used, and a region of -1.5 kb (upstream) and +20 bp (downstream) from the transcription start site was analyzed.

Genes family	*Name of Gene in N. tabacum*	*Gene identity in N. tabacum*	Sequence	Position	DNA strand
GSTs	▪ *GSTF6-like*	107794811	CAAAGATATC	522<br>1167	-<br>+
	▪ *GSTF8-like*	107795177	CTAAATGAACT<br>CAAATTAATCA	140<br>1070	-<br>+
	▪ *GSTZ1-like*	107828152	CAAAGATATC	1119<br>1466	+<br>+

Table S3. Primer list used for quantitative real-time PCR expression studies of genes potentially involved in metabolism of xenobiotic compounds. These were designed using the website https://www.idtdna.com/scitools/Applications/RealTimePCR/Default.aspx.

Genes family	***N. tabacum* Gene/identity**	**Primer sequence 5´ 3´ Forward (F) and reverse (R)**	**Length of primers (bp)**	**PCR product size (bp)**
GSTs	▪ *GSTF6-like* ID107794811	**F** TGTGAAGGATTGGTGTGCTG<br>**R** ACAACTATTACGCCTCAATCCC	20<br>22	115
	▪ *GSTF8-like* ID107795177	**F** TCGAGAAGGAGCTGGATTTTG<br>**R** GGATTGAGGGAAAGGTAAGGG	21<br>21	85
	▪ *GSTZ1-like* ID107828152	**F** TCTGTTGAGGGTATTTGAGGC<br>**R** GCAGGTATGTCAACGGTTTG	21<br>20	148
Clock genes	▪ *NtLHY* ID107775222	**F** CACTCTTTTCAAGGAAGGTG<br>**R** GTCGAAGGTGTTACAAGAGC	20<br>20	241
	▪ *NtTOC1* ID107818975	**F** ATCGTAGAACGGCAGCACTT<br>**R** TCACAAACTGTCCCCTCACA	20<br>20	129
Reference gene	▪ *NtEF1* ID107791623	**F** TGAGATGCACCACGAAGCTC<br>**R** CCAACATTGTCACCAGGAAGTG	20<br>22	51
